# Prior cocaine self-administration impairs attention signals in anterior cingulate cortex

**DOI:** 10.1038/s41386-019-0578-2

**Published:** 2019-11-27

**Authors:** Daniela Vázquez, Heather J. Pribut, Amanda C. Burton, Stephen S. Tennyson, Matthew R. Roesch

**Affiliations:** 1 Department of Psychology, University of Maryland, College Park, MD 20742 USA; 2 Program in Neuroscience and Cognitive Science, University of Maryland, College Park, MD 20742 USA

**Keywords:** Neuronal physiology, Cognitive neuroscience, Diseases of the nervous system

## Abstract

Although maladaptive decision-making is a defining feature of drug abuse and addiction, we have yet to ascertain how cocaine self-administration disrupts neural signals in anterior cingulate cortex (ACC), a brain region thought to contribute to attentional control. To address this issue, rats were trained on a reward-guided decision-making task; reward value was manipulated by independently varying the size of or the delay to reward over several trial blocks. Subsequently, rats self-administered either a cocaine (experimental group) or sucrose (control) during 12 consecutive days, after which they underwent a 1-month withdrawal period. Upon completion of this period, rats performed the previously learned reward-guided decision-making task while we recorded from single neurons in ACC. We demonstrate that prior cocaine self-administration attenuates attention and attention-related ACC signals in an intake-dependent manner, and that changes in attention are decoupled from ACC firing. These effects likely contribute to the impaired decision-making—typified by chronic substance abuse and relapse—observed after drug use.

## Introduction

Difficulties in treating addiction arise from vulnerability toward cue-induced cravings that lead to relapse and reinstatement of drug-seeking behavior [1–4]. Treating addiction is further complicated by drug-induced impairments of circuits that are critical for behavioral control and contribute to functions imperative for decision-making. Thus, addicts have the onerous task of having to overcome withdrawal symptoms and cravings without the aid of fully functioning circuits that contribute to optimal behavior.

The anterior cingulate cortex (ACC) has been implicated in a number of cognitive functions, including conflict monitoring and detection, arousal, surprise, feedback and error processing, reward predictions, perceptual decision-making, prediction errors, and attentional control [5–21]. Across studies, the ACC contributes to behavioral adjustments that are triggered by the occurrence of unexpected events, and plays a key role in shifting the allocation of attentional resources toward behaviorally relevant stimuli when there are violations in outcome expectancies, uncertainty, or conflict between competing stimuli or behaviors [7, 8, 11, 13, 14, 16–19].

In line with these theories, we previously reported that ACC firing correlates with Pearce and Hall-like changes in attention that occur during learning [16, 22]. Specifically, behavioral measures of attention were correlated with increases in ACC firing on trials following unexpected outcomes (i.e., unsigned prediction errors). Furthermore, changes in ACC neural firing occurred prior to and during the processing of trial events, as rats adapted to new behavior–outcome contingencies [16]. Here, we explored whether shifts in behavior and associated neural correlates in the ACC were impacted by prior cocaine use.

To address this issue, we recorded from single neurons in rat ACC while rats performed a two-choice reward-guided decision-making task [16]. Recordings took place following 12 consecutive days of self-administration and a month-long withdrawal period, in order to assess the neural and behavioral long-term effects of cocaine [23, 24]. Optimal task performance required rats to detect unexpected changes in reward value and update behavior accordingly to select the more favorable reward outcome on free-choice trials, while maintaining accurate responding on forced-choice trials. Rats that self-administered cocaine exhibited a stronger behavioral bias toward immediate reward, were faster at responding, and performed worse on forced-choice trials. Here, we demonstrate that violation-induced behavioral adaptations and related ACC firing are impaired following cocaine self-administration.

## Materials and methods

### Subjects

Male and female Long–Evans rats (*n* = 18; 16 M, 2 F) were obtained at ~2–3 months of age from Charles River Laboratories, weighing in the range of 150–200 g. Rats were tested at the University of Maryland (UMD), College Park in accordance with UMD and NIH guidelines. During behavioral testing, food was available ad libitum; water intake was restricted to ensure motivation for task performance.

### Experimental design

**Fig. 1:**
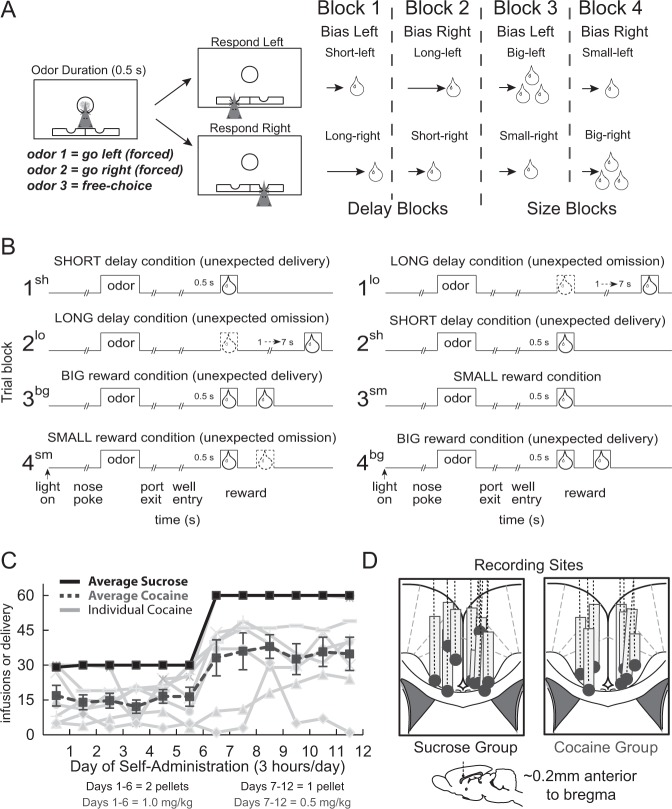
Reward-guided decision-making task, variability in self-administration, and recording sites. **a**, **b** Schematics of the reward-guided decision-making task, where value from block to block (60 trials per block) was manipulated by altering delay to (short (0.5 s) or long (1–7 s); blocks 1–2) or size of (large (one bolus) or small (two boli); blocks 3–4) liquid sucrose reward. **a** Schematic represents general task structure, block sequence, and odor panel layout. On each trial, rats nose-poked into an odor port to receive one of three odor cues, and then responded in the corresponding fluid well to receive reward. One odor signaled reward in the left well (forced choice), another indicated reward in the right well (forced choice), and a third odor signaled reward at either well (free choice). Odors were counterbalanced. **b** Schematic illustrates trial structure (i.e., houselights to reward delivery) and emphasizes shifts in value that occur during the task that are only cued by receiving unexpectedly the reception of better or worse reward at the start of trial blocks. **c** Average number of active lever presses per day during the 12 days of self-administration, for both cocaine (*n* *=* 7) and control (*n* *=* 11) rats. **d** Location of recording sites verified by histology (Paxinos and Watson). Gray boxes mark the extent of the recording locations.

All rats were trained for 6 weeks on a reward-guided decision-making task (Fig. 1; for more detail, see ref. 23). On each trial, nose poke into the odor port following house-light illumination resulted in delivery of a directional odor cue. One odor instructed the rat to go to the left fluid well to receive reward (forced choice), a second odor instructed the rat to go to the right fluid well to receive reward (forced choice), and a third odor indicated that the rat could obtain reward at either well (free choice). On forced-choice trials, if the rat went to the incorrect well, reward was not delivered. Odors were presented in a pseudorandom sequence and were counterbalanced across rats.

At the start of each session, one well was randomly assigned to have a short delay to reward (0.5 s), and the other a long delay (1–7 s) (Fig. 1a, b: Block 1). In the second block of trials, these contingencies were interchanged (Fig. 1a, b: Block 2). The length of the delay under “long” conditions abided by the following algorithm—the side designated as long started with a delay of 1 s, and increased by 1 s every time that side was chosen by the rat during a free-choice odor trial (maximum of 7 s). The delays on forced-choice trials were yoked to the delay on free-choice trials. Intertrial intervals were normalized so that the total trial length was the same across short- and long-delay trial types (i.e., if the delay increased by 1 s on long-delay trials, the ITI on short-delay trials increased by 1 s). During the final two blocks of the task, the delay preceding reward delivery was held constant (0.5 s on both sides) while manipulating the size of the expected reward (Fig. 1a, b: Blocks 3 and 4). Throughout the task, reward consisted of a single 0.05-ml bolus of 10% sucrose solution; large reward consisted of an additional bolus being delivered 0.5 s after the first bolus. At least 60 trials per block were collected for each session.

Following training, all rats underwent a surgical procedure during which an intravenous catheter (Dow Corning Silastic tubing) was inserted into the right jugular vein [14], and a drivable chronic electrode (8 microwires; 27-G cannula) was implanted into the ACC (0.2 mm anterior to bregma, ±0.5 mm lateral, and 1 mm ventral to brain; 16, 23). After 1 week of recovery, the randomly assigned experimental (*n* = 7; 6 M, 1 F) and control (*n* = 11; 10 M, 1 F) groups engaged in cocaine or sucrose self-administration for 12 consecutive days, after which they underwent a month-long withdrawal period (for further detail, see refs. 23, 24). Rats lever-pressed to receive either cocaine or sucrose on a fixed-ratio schedule. After each active lever press, a cue light was illuminated for 2.3 s—the duration of each cocaine infusion. Following each lever press, a 20-s time-out period occurred during which rats were unable to lever-press for reward.

During the first 6 days of cocaine self-administration, each intravenous cocaine infusion was 1.0 mg/kg (maximum 30 infusions or 3 h). The infusion dose during the final 6 days was reduced to 0.5 mg/kg (maximum 60 infusions or 3 h). This procedure allowed us to assess increases in drug-seeking behavior when doses are cut into half to maintain the desired level of drug intake. Continuous access to high cocaine doses evokes drug-taking and drug-seeking behaviors that are consistent with promoting symptoms of addiction [25] and has been shown to change behavior and neural signals in other brain regions [23, 24]. Further, individual rat variability in lever pressing parallels individual differences observed in human cocaine consumption [26]. The control group followed the same protocol with the same parameters delineated above, receiving two sucrose pellets per lever press during days 1–6, and only one per active lever press for days 7–12. Recordings (Plexon) during task performance began 1 month after self-administration [23, 24]. Electrodes were advanced 40 μ daily.

Our previous work demonstrated that light-on latencies (house-light illumination to odor port nose poke) were significantly faster at the beginning compared with the end of trial blocks; further, ACC firing increased during early trials and was negatively correlated to light-on latencies [16]. Here, we replicate this analysis by examining light-on latencies and firing rates during early (first ten) and late trials (last ten) for each trial type within a block. Other analyses break trials down into five trial bins to better display time course. Both light-on latencies and firing-rate indices were computed to capture differences between early and late activity in each trial block (early − late/early + late). Firing rate was taken from house-light onset to completion of the response for correct trials only. Wilcoxon tests were used to measure significant shifts in the distribution of indices from zero, and to determine differences between control and cocaine-exposed groups (*p* < 0.05).

### Behavioral analysis

Behavior in the recording task was analyzed by calculating the percent of correct responses on forced-choice trials (the amount of trials the animal correctly responded to the side corresponding to the directional odor cue), the percent of trials rats chose a particular valued condition (short, long, large, and small) on free-choice trials, and reaction times (odor offset to odor port exit). Calculations were split into the first and last ten trials for each trial type. We have previously shown that analyzing ten trials from each trial type captures the development of learning at the start of trial blocks, and provides a large enough sample to conduct behavioral and neural statistics [23, 24]. Free-choice reaction times were not split into early and late trials due to lower proportions of trials (e.g., fewer low-value choices late in the trial block). Behavioral analyses were computed for each individual session (separated by cocaine and control groups), and then averaged across sessions for each group. Conducting analyses across sessions—instead of across individual subjects—provides a better reflection of the neural correlates corresponding to behavior. Importantly, the main behavioral findings described in this paper have been replicated in three different studies. Multifactor analysis of variance (ANOVA; factors included group (sucrose vs. cocaine), reward value (high vs. low), value manipulation (size vs. delay), and phase of learning (early: first ten trials vs. late: last ten trials per trial type)) and *t* tests (*p* < 0.05) were used to determine differences between the cocaine and control group trials per trial type.

## Results

### Self-administration

All rats were trained on the reward-guided decision-making task (Fig. 1a, b) prior to implantation of electrodes in ACC (Fig. 1d) and intravenous catheters for cocaine self-administration. Rats self-administered sucrose pellets (*n* = 11) or cocaine (*n* = 7) over the course of 12 days. During days 1–6 (1 mg/kg of cocaine or 2 sucrose pellets per lever press), the average number of infusions or pellet deliveries across rats out of a maximum of 30 was 18.2 (±9.2 standard deviation (s.d.)) and 29.9 (±0.4 s.d.) for cocaine and sucrose, respectively (Fig. 1c). During days 7–12 (0.5 mg/kg cocaine or 1 sucrose pellet per press), the average number infusions or pellet deliveries across rats out of a maximum of 60 was 37.9 (±16.0 s.d.) and 60 (±0 s.d.) for cocaine and sucrose, respectively. Figure 1c illustrates the infusions and pellet deliveries across the 12 days for sucrose and cocaine groups. In addition, infusions for individual rats that self-administered cocaine are displayed (gray) to convey variability.

### Cocaine made rats more sensitive to reward delays, worsened task performance, and accelerated reaction times

**Fig. 2:**
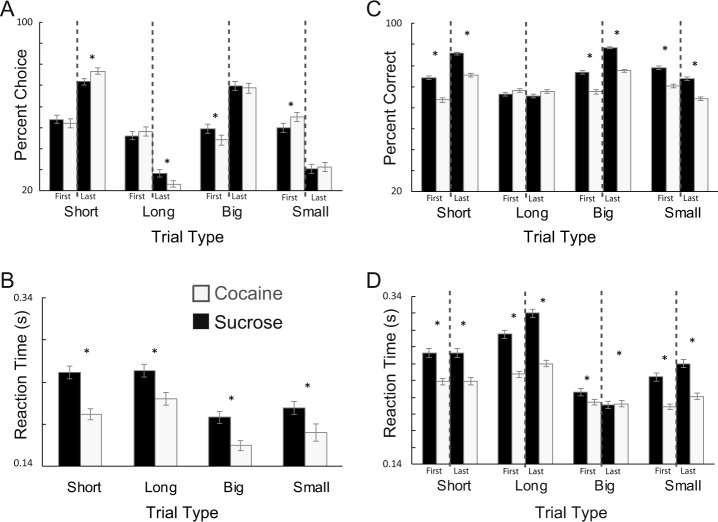
Cocaine made rats more sensitive to delays, worsened performance, and accelerated reaction times. **a** Percent choice on free-choice trials in each value manipulation over the first ten and last ten trials of each block. **b** Reaction time (odor port exit minus odor offset) on all free-choice trials for each value manipulation. **c** Percent correct on forced-choice trials in the same manner as **a**. **d** Reaction time (odor port exit minus odor offset) on forced-choice trials. For these analyses, behavior was analyzed by session (controls, black bars; cocaine, gray bars). Error bars indicate SEM. Asterisks indicate significance (*p* < 0.05) in multifactor ANOVA and/or post hoc *t* tests. For the cocaine group, *n* = 7; control group, *n* = 11.

Replicating our previous results, rats exhibited bias toward higher-value rewards during delay blocks [23, 24]. In an ANOVA with percent choice as the dependent variable, there was a significant main effect of reward value (*F*(1,2658) = 2781.3, *p* < 0.01; *d* = 2.4), as both control and cocaine rats preferred high-value reward (short; large; Fig. 2a). There was a significant interaction between group, value, and block phase (*F*(1,2658) = 415.0, *p* < 0.001), with cocaine rats choosing high-value reward significantly more often than controls in the last ten free-choice trials during delay manipulations (Fig. 2a; (*t*_(1197)_ = 2.08, *p* < 0.05); *d* = 0.26). Furthermore, cocaine rats were significantly faster at responding on all free-choice trial types compared with controls (Fig. 2b; ANOVA; main effect of group (*F*(1,2630) = 6.55, *p* < 0.05; *d* = 1.3). Although cocaine made rats more sensitive to delay manipulations, cocaine and control rats chose large over small reward at similar rates (Fig. 2a; (*t*_(1197)_ = 0.19, *p* = 0.85); *d* = 0.06).

In ANOVAs with percent correct and reaction time on forced-choice trials as the dependent variables, we found a main effect of value (percent correct: *F*(1,2658) = 228.2, *p* < 0.001; forced-choice reaction time: *F*(1,2658) = 16.84, *p* < 0.01) and an interaction of value and phase (percent correct: *F*(1,2658) = 144.99, *p* < 0.01; *d* for percent correct = 0.15; *d* for reaction time = 2). This indicates that overall, both control and cocaine rats were significantly better and faster on high-value forced-choice trials, particularly in the late phase of each block (Fig. 2c). However, there was also a main effect of group in the ANOVAs on forced-choice behavioral measures (percent correct: *F*(1,2658) = 185.80, *p* < 0.001; *d* = 0.53; forced-choice reaction time: *F*(1,2658) = 112.54, *p* < 0.001; *d* = 0.12), with cocaine rats being significantly faster and worse on forced-choice trials compared with controls (Fig. 2c, d). Notably, the majority of these behavioral measures were correlated with the number of cocaine infusions and the differential drug seeking following dosage reduction. We found that both were negatively correlated with a percent correct on forced-choice trials (infusions vs. percent correct: *p* < 0.001, *r*^2^ = 0.08; difference between weeks 1 and 2 vs. percent correct: *p* < 0.001, *r*^2^ = 0.09) and were positively correlated with a response bias toward more immediate reward on free-choice trials (infusions vs. percent correct: *p* < 0.05, *r*^2^ = 0.030; difference between weeks 1 and 2 vs. percent correct: *p* < 0.05, *r*^2^ = 0.034). Last, there was a significant negative correlation between reaction times on forced-choice trials and increases in cocaine seeking during week 2 (difference between weeks 1 and 2 vs. percent correct: *p* < 0.05, *r*^2^ = 0.037).

We conclude that previous cocaine self-administration had a long-term impact on behavior during performance of the reward-guided decision-making task. Overall, cocaine rats exhibited stronger response biases toward more immediate reward on free-choice trials, and were significantly faster and worse on forced-choice trials. These results are consistent with previous work demonstrating that cocaine self-administration makes rats more impulsive during delay tasks [24, 27–34].

### Cocaine self-administration attenuates attentional signals

**Fig. 3:**
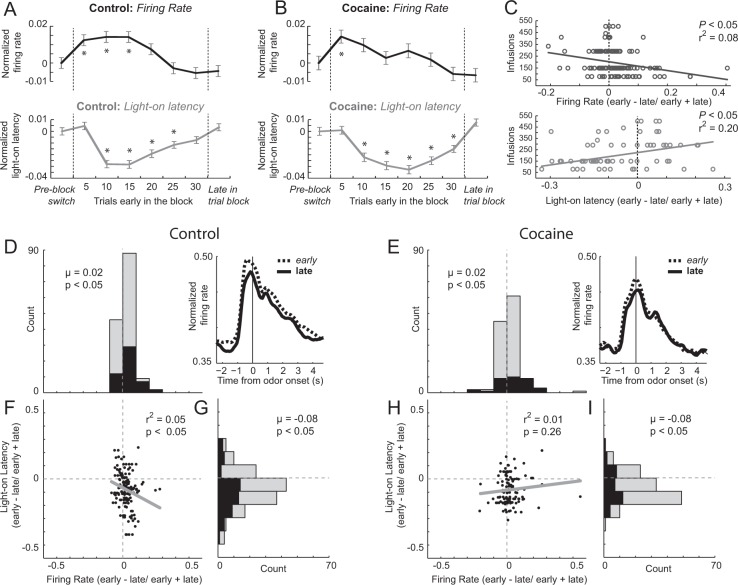
Cocaine attenuates cognitive control signals in ACC. **a**, **b** Normalized firing rates (black; epoch = houselights on to well entry) and light-on latencies (gray; latency to enter odor port upon house-light illumination) for the average of the last five trials in a block before a switch in contingencies (“pre-block switch”), the trials post switch in five trials bins, and the average of the last five trials in the current trial block (“late in trial block”) for control (**a** *n* = 145 cells; 11 rats) and cocaine-exposed (**b** *n* = 123 cells; 7 rats) rats. Firing rates and light-on latencies are normalized to the max values within each session, averaged, and then subtracted from “Pre-block switch” trials so that comparisons can be made across groups. Asterisks denote significant differences (*t* test; *p* < 0.05) from pre-block switch trials. **c** Correlation between firing-rate indices (black) and total number of infusions during cocaine self-administration and between light-on indices (gray) and total number of infusions during cocaine self-administration. Firing rate and light-on latency indices were computed by subtracting late and early trials (last ten trials) from each trial type (first ten trials) and dividing by their sum (early − late/early + late). **d**, **e** Distributions of firing-rate indices for controls (**d**) and cocaine-exposed (**e**) rats. Black bars represent cells with firing that differed significantly for early compared with late trials (*t* test; *p* < 0.05). Insets to the right of each panel illustrate normalized average firing during early (dashed) and late (solid) trials averaged over all trial types. Firing is aligned odor onset. Nose poke into the odor port occurred 500 ms prior to odor presentation and was triggered by illumination of the houselights. Port exit occurred roughly 750 ms after odor onset (500 ms of odor presentation +~250 ms of reaction time). **f** The correlation between firing-rate indices (**d**) and light-on latency indices (**g**) for control rats. **h** The correlation between firing-rate indices (**e**) and light-on latency indices (**i**) for cocaine-exposed rats. Black bars in **g** and **i** represent sessions where light-on latencies significantly differed between early and late trials (*t* test; *p* < 0.05). Wilcoxon tests were used to measure significant shifts in the distribution of indices from zero, and to determine differences between control and cocaine-exposed groups (*p* < 0.05).

Previously, we have shown that attention and firing in ACC are elevated during early trials when rats are updating action–outcome contingencies. One measure of attention is the latency at which rats respond to external stimuli that signal initiation of behavioral trials. Here, we examine how quickly rats nose-poke into the central port upon illumination of houselights—referred to as light-on latency. Latency to approach the odor port precedes any knowledge of the upcoming reward; thus, this measure cannot reflect the nature or evaluation of the reward to be received at the end of the trial. Further, this measure cannot reflect a reduction in motivation over the course of the session, because latencies are significantly shorter on early trials within a block of trials, irrespective of the amount of preceding trials [16]. Faster light-on latencies are thought to reflect accelerated processing of trial events (e.g., cues, responses) as rats increase their reception of unexpected shifts in reward contingencies [35–37]. Light-on latencies might also reflect an investigatory reflex, or a reengagement of instrumental task performance (i.e., increased cognitive control). A similar phenomenon has been described during recovery from habituation following shifts in learned contingencies, mirroring theoretical changes in Pearce and Hall models of attention [35–40]. A replication of this effect is illustrated in Fig. 3a, b for controls and rats exposed to cocaine. In this figure, light-on latencies averaged across five trial bins were normalized to latencies just prior to block transitions (“pre-block switch”; last five trials in a block of trials). Consistent with previous reports, light-on latencies became faster several trials after the block transition before returning to pre-block switch levels (Fig. 3a, b (gray); five pre-block switch trial bin vs. second bin of five trials post switch (*t* test; *control*: *t*(144) = 4.80; *p* < 0.0001; *cocaine*: *t*(122) = 3.64; *p* < 0.0001)).

To determine the impact that block transitions had on neural firing, we examined neurons that increased firing from house-light onset (i.e., trial start) to well entry (i.e., completion of the behavioral response) averaged across all rewarded trials and trial types. Neurons that exhibited general increases in firing during the trial were previously reported to increase after violations in reward expectancies. [16]. Overall, 145 (20%) and 123 (21%) of ACC neurons increased firing during the “trial epoch” compared with baseline (1 s preceding light onset; Wilcoxon; *p* < 0.05) in control and cocaine-exposed rats, respectively. For both groups, the proportion of neurons that increased firing during the trial epoch was significantly higher than expected from chance alone (control: *χ*^2^ = 332; *p* < 0.05; *cocaine*: *χ*^2^ = 319; *p* < 0.05) and the frequency of counts relative to the total sample did not significantly differ between groups (*χ*^2^ = 0.26; *p* = 0.61).

As previously reported, we found that ACC activity was stronger at the beginning compared with the end of each trial block (Fig. 3a, b, black). As with light-on latencies, firing-rate changes in ACC were significantly different early in trial blocks. However, unlike changes in light-on latencies, firing-rate changes were significantly different within the first block of five trials (5 pre-block switch trial bin vs. first bin of five trials post switch; *t* test; control: *t*(144) = 2.32; *p* = 0.02; cocaine: *t*(122) = 2.41; *p* = 0.02). Thus, changes in firing preceded changes in light-on latencies (Fig. 3a, b; black vs. gray).

To further quantify changes from the beginning and end of trial blocks, we computed an index that captured differences between early (first ten trials) and late (last ten trials) trials for each trial type for each neuron in both control and cocaine groups (index = early − late/early + late). For light-on latencies, we observed significant shifts below zero for both groups (indicating faster latencies early in the trial blocks), with no difference between them (Wilcoxon: control (Fig. 3g): *n* = 145; *μ* = −0.08; *p* < 0.0001; cocaine (Fig. 3i): *n* = 123; *μ* = −0.08; *p* < 0.0001; control vs. cocaine: *z* = 0.72; *p* = 0.47). Likewise, for distributions of firing-rate indices, we found significant shifts above zero for both groups—indicative of higher firing at the beginning of trial blocks—with no significant difference between groups (Wilcoxon: control (Fig. 3d): *n* = 145; *μ* = 0.23; *p* < 0.0001; cocaine (Fig. 3e): *n* = 123; *μ* = 0.22; *p* < 0.0001; control vs. cocaine: *z* = 1.04; *p* = 0.30).

At the single-neuron level, firing of 50 (34%) and 24 (20%) of neurons were significantly modulated during the first compared with the last ten trials in control and cocaine-exposed rats, respectively (Fig. 3d, e, black bars; *t* test; *p* < 0.05). This difference between groups approached significance (*χ*^2^ = 3.76; *p* = 0.053). Of these neurons, 38 and 12 neurons from control animals were significantly higher and lower during the first compared with the last ten trials (Fig. 3d, black bars; *t* test; *p* < 0.05). For the cocaine group, firing of 22 and 12 neurons exhibited significantly higher and lower firing during early compared with late trials, respectively (Fig. 3e, black bars; *t* test; *p* < 0.05). Only for controls did the counts of neurons that exhibited significantly higher firing during early trials significantly outnumber those showing significantly lower firing (control: *χ*^2^ = 13.41; *p* < 0.05; cocaine: *χ*^2^ = 1.44; *p* = 0.09); however, the frequency of neurons did not differ significantly between groups (*χ*^2^ = 0.77; *p* = 0.38).

In summary, for both groups of rats, we found that early increases in ACC firing preceded faster light-on latencies—suggesting that the two processes were related, and that ACC might be contributing to changes in attention that occur following unexpected shifts in reward contingencies. Consistent with our previous work, we show that ACC firing and light-on latencies are correlated in control rats. In control rats, the two indices were negatively correlated (Fig. 3f, *p* *=* 0.009*, r*^2^ *=* 0.05), suggesting that during sessions in which ACC firing rates were higher, rats were more attentive. Interestingly, in cocaine rats, the correlation was positive but not significant (Fig. 3h, *p* = 0.26; *r*^2^ = 0.01), and was significantly different than controls (Fisher r-to-z transformation; *z* = 2.64; *p* = 0.008), suggesting that attentional control was decoupled from firing in ACC after cocaine exposure.

Next, we explored whether these changes were impacted by the amount of cocaine rats self-administered as measured by the total amount of cocaine infused and the degree to which rats increased drug seeking from weeks 1 to 2 during self-administration. Remarkably, we found a positive correlation between light-on latencies and both measures of self-administration (infusions vs. light-on latency indices (Fig. 3c, gray): *p* < 0.0001; *r*^2^ = 0.20; difference between weeks vs. light-on latency indices: *p* = 0.001; *r*^2^ = 0.11). The opposite relationship was observed between firing-rate indices and both measures of self-administration (i.e., negative correlation; infusions vs. firing-rate indices (Fig. 3c, black): *p* = 0.002; *r*^2^ = 0.08; difference between weeks vs. firing-rate indices: *p* = 0.0009; *r*^2^ = 0.08). Thus, rats that had self-administered more cocaine exhibited reduced attention and attention-related firing early in trial blocks.

### Signals related to both up- and downshifts in value were impacted by cocaine

**Fig. 4:**
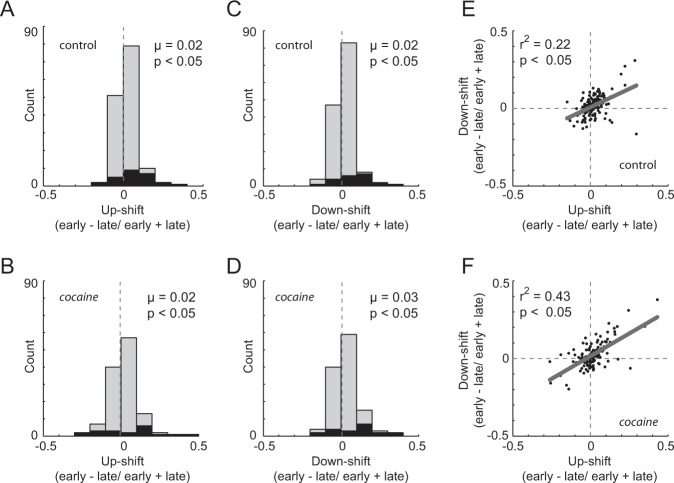
Neural signals related to both up- and downshifts in value were impacted by cocaine. Firing-rate indices for upshifts (i.e., shift to short delay or large reward); **a**, **b** and downshifts (i.e., shift to long delay or small reward); **c**, **d** were computed by subtracting late trials (last ten trials) from early trials (first ten trials) for each shift type and dividing by the sum (early − late/early + late) for control (**a**, **c**; *n* = 145; 11 rats) and cocaine-exposed (**b**, **d**; *n* = 123; 7 rats). Black bars represent cells whose firing was significantly different for early compared with late trials (*t* test; *p* < 0.05). **e**, **f** Correlation between up- and downshift firing-rate distributions for controls (**e**) and cocaine-exposed (**f**) rats.

In a final neural analysis, we investigated whether observed drug-induced changes in firing rates were more or less pronounced after up- versus downshifts in value. Recall that block shifts were only cued by rats experiencing unexpected increases (i.e., larger or more immediate—upshifts) or decreases (i.e., smaller or delayed—downshifts) in reward value. One tenant of the Pearce and Hall model of attention is that signals increase following both types of value shifts. Indeed, we have shown that firing-rate changes in ACC exhibit unsigned increases in activity to both up- and downshifts in value [6]. Here, we replicate this effect in ACC for both control and cocaine-exposed rats. Firing-rate indices (early − late/early + late) were significantly shifted in the positive direction for both up- and downshifts in value (Wilcoxon: control: upshift (Fig. 4a): *μ* = 0.02; *p* < 0.0001; downshift (Fig. 4c): *μ* = 0.02; p = 0.002; cocaine (Fig. 4b): upshift: *μ* = 0.02; *p* = 0.006; downshift (Fig. 4d): *μ* = 0.03; *p* = 0.001). Further, up- and downshift indices were positively correlated with each other, demonstrating that single neurons exhibited similar increases in firing following either an up- or downshift in value (control (Fig. 4e): *p* < 0.0001; *r*^2^ = 0.22; cocaine (Fig. 4f): *p* < 0.0001; *r*^2^ = 0.44). Last, both up- and downshift distributions were negatively correlated with the cocaine infusions (upshifts: *p* = 0.01; *r*^2^ = 0.05; downshifts: *p* = 0.003; *r*^2^ = 0.07) and increases in drug seeking during week 2 during self-administration (upshifts: *p* = 0.007; *r*^2^ = 0.06; downshifts: *p* = 0.0007; *r*^2^ = 0.10).

To further illustrate these correlations and to better examine variability within our sample, we divided data rats into high and low intake [41]. Two rats in particular (Fig. 1c: diamonds; triangles) exhibited significantly lower drug intake and drug seeking relative to the other rats that had self-administered cocaine. Both of these rats pressed significantly less than each of the other rats across the 12 days of self-administration (*t* tests, *t*(22) > 5.47; *p* < 0.009). Distributions of firing-rate indices from these two rats (*n* = 2 rats; 38 sessions; 80 neurons) were significantly shifted in the positive direction following upshifts in value (Wilcoxon; *μ* = 0.034; *p* < 0.0001); control and low-intake distributions were not significantly different from each other (Wilcoxon; *z* = 1.24; *p* = 0.22). On the other hand, distributions of firing-rate indices for rats that self-administered more cocaine (*n* = 5 rats, 28 sessions; 43 neurons) were not significantly shifted (Wilcoxon; *μ* = −0.009; *p* = 0.788); high- and low-intake distributions were significantly different from each other (Wilcoxon; *z* = 2.63; *p* = 0.008), as were differences between controls and high-intake distributions (Wilcoxon; *z* = 2.05; *p* = 0.04). Although there were correlations with intake, firing-rate distributions for downshifts did not significantly differ between control, high-intake, and low-intake groups (Wilcoxon; control vs. high: *z* = 1.48; *p* = 0.14; control vs. low: *z* = 0.48; *p* = 0.63; high vs. low: *z* = 0.72; *p* = 0.47).

### Relationship between prediction errors, light-on latencies, and task performance

**Fig. 5:**
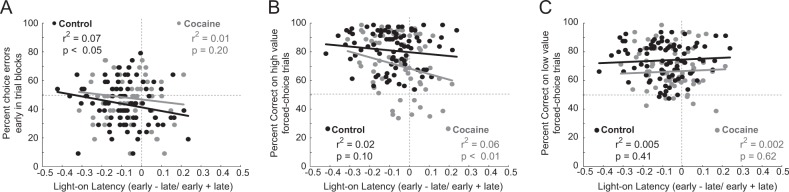
Relationship between light-on latencies and task performance. **a** Correlation between light-on latency indices and choice errors during the first 20 trials (10 per trial type) of delay blocks. **b** Correlation between light-on latency indices and percent correct average over forced-choice high-value trials (i.e., short delay and large reward) during the first ten trials of each trial type. **c** Correlation between light-on latency indices and percent correct average over all forced-choice low-value trials (i.e., long delay and small reward) during the first ten trials of each trial type. Control = black; Cocaine = gray.

During performance of this task, we posit that reward prediction errors trigger changes in attention during subsequent trials. This suggests that the strength of attentional modulation should be correlated with the proportion of prediction errors (i.e., errant choices) occurring at the start of each trial block. Figure 5a plots the light-on latency distributions described above (Fig. 3d, e) against the percentage of choice error trials early during delay blocks (i.e., first ten trials; Fig. 2a). In both control and cocaine-exposed rats, there was a negative correlation between light-on latencies and free-choice behavior (Fig. 5a; control (black): *n* = 145; *p* = 0.0003; *r*^2^ = 0.06; cocaine (gray): *n* = 123; *p* = 0.19; *r*^2^ = 0.01), with no differences in the strength of the correlation between the two groups (Fisher r-to-z transformation; *z* = 1.09; *p* = 0.28). Next, we determined whether light-on latency indices were correlated with forced-choice performance. Faster latencies at the beginning of trial blocks—when reward contingencies had recently been reversed—were associated with better forced-choice performance on high-value trials, with no differences between groups (Fig. 5b; high value; control: *p* = 0.098; *r*^2^ = 0.02; cocaine: *p* = 0.009; *r*^2^ = 0.05; Fisher r-to-z transformation: *z* = 0.82; *p* = 0.412; Fig. 5c; low value; control: *p* = 0.409; *r*^2^ = 0.005; cocaine: *p* = 0.618; *r*^2^ = 0.002; Fisher *r*-to-*z* transformation: *z* = 0.19; *p* = 0.849). Overall, these results suggest that increased attention following reward prediction errors contributes to better performance on high-value trials in both control and cocaine-exposed rats.

## Discussion

In summary, we show that neural signals related to attentional control in ACC are attenuated following cocaine exposure, and that neural firing and behavioral changes were correlated with the degree to which rats self-administered cocaine. Altered attentional control observed after cocaine exposure potentially impacts decisions that require increased neural processing in the face of competing information, uncertainty, or expectancy violations, likely contributing to impaired decision-making that leads to drug use and relapse.

Here, we have used light-on latencies as a measure for when and how strongly attentional control processes are engaged. During performance of our task, we think that faster light-on latencies are thought to reflect accelerated processing of trial events (e.g., cues, responses) after unexpected shifts in reward contingencies [35–37], serving to increase attention during investigation of new contingencies and task reengagement when habitually learned block structures must be updated following unexpected increases or decreases in reward value, mirroring theoretical changes in Pearce and Hall models of attention [35–40]. Consistent with this observation, the percent errors that occurred early in trials blocks were correlated with changes in light-on latencies. We also think that ACC contributes to maintaining attention to cues as block adjustments are occurring, allowing rats to follow forced-choice rules while adapting action–outcome contingencies during free-choice trials. This idea fits with the observation that when attention was low, rats tended to perform worse on high-value forced-choice trials.

Overall, neural and behavioral changes observed after cocaine self-administration were maladaptive. Cocaine rats were more impulsive in that they exhibited a behavioral bias toward more immediate over delayed reward, and performed significantly worse on forced-choice trials. As a result, cocaine-exposed rats acquired less reward overall—due to the nondelivery of reward on incorrect forced-choice trials, and because short-delay trials were normalized so that overselection of more immediate reward did not result in greater reward over multiple trials. Altered ACC function would likely impact impulsivity and cognitive control as measured in other tasks, such as the stop-signal task, known to modulate ACC firing in rats [42] and is impacted in addiction [43, 44].

Although it is difficult to know the exact homolog of human ACC, rat ACC connectivity does overlap with primate ACC [45], and sits in a prime position to mediate behavioral control via its monosynaptic projections to dorsal striatum [46, 47], subthalamic nucleus [47], prefrontal cortex [48], and locus coeruleus [49]. Further, our recordings are consistent with signals reported in primate ACC [17], and firing of single neurons in the same region of rat ACC are elevated during response conflict, as reported in humans [42]. This work is also consistent with research in humans examining cocaine-induced impairments in attention and cognitive control [50–53]. Thus, our work likely translates well to the human condition, providing additional evidence of drug-induced changes in brain and behavior at the level of single neurons [54] while controlling for confounding variables that might influence work in humans (e.g., genetics, life history, and polysubstance abuse).

These findings provide a new dimension to the growing number of problems that arise after drug use, paralleling changes in striatal and dopamine circuits that give rise to reward predictions and signed prediction errors. It is already known that neural signals in nucleus accumbens core (NAc), dorsolateral striatum (DLS), and ventral tegmental area (VTA), dopamine (DA) neurons are abnormal following cocaine self-administration [34, 55–57]. Cocaine disrupts NAc’s ability to encode reward predictions and expectancies during delays preceding reward delivery [23]. It also impairs signed prediction error signals generated by VTA–DA neurons, and alters encoding in DLS, in line with elevated response biases on free-choice trials [23, 58]. We suspect that artificially stimulating ACC in cocaine-exposed rats will repair many of these problems, via mechanisms that focus neural resources to task events following expectancy violations—leading to better prediction and error encoding in both NAc and VTA–DA neurons, and stronger cognitive control over DLS. As a result, we predict that ACC stimulation during decision-making following events that are uncertain, unexpected, or incongruent with competing information may translate to improved cognitive control in animals that have been previously exposed to drugs of abuse.

## Funding and disclosure

This work was supported by the National Institutes of Health National Institute on Drug Abuse Grant R01 DA031695. The authors declare no competing interests.
